# Effectiveness of a Mindfulness-Based Mobile Application for the Treatment of Depression in Ambulatory Care: Protocol for a Randomized Controlled Trial

**DOI:** 10.2196/33423

**Published:** 2022-03-31

**Authors:** Jan Sarlon, Jessica P K Doll, Aline Schmassmann, Serge Brand, Naomi Ferreira, Markus Muehlhauser, Stefanie Urech-Meyer, Nina Schweinfurth, Undine Emmi Lang, Annette Beatrix Bruehl

**Affiliations:** 1 University Psychiatric Clinics University of Basel Basel Switzerland; 2 Department of Sport, Exercise and Health Faculty of Medicine University of Basel Basel Switzerland; 3 Sleep Disorders Research Center Kermanshah University of Medical Sciences Kermanshah Iran

**Keywords:** depression, mindfulness, mhealth, ehealth, stress level

## Abstract

**Background:**

Patients with major depressive disorder (MDD) often experience relapses despite regular treatment with pharmacotherapy and psychotherapy. Further, long waiting lists and more demand than treatment capacity characterize ambulatory settings. Mindfulness-based interventions proved to be effective in relapse prevention in MDD. Next, mindfulness-based interventions in the form of free mobile applications can be an effective augmentation of the treatment as usual and can fill a gap in ambulatory care.

**Objective:**

Given this background, the aim of this randomized controlled study is to assess the effectiveness of additional MBI via a mobile app on the symptom severity and stress levels, compared to treatment as usual.

**Methods:**

A total of 140 individuals with MDD will be randomly allocated to the intervention or control condition. The intervention consists of the daily use of the mindfulness mobile application Headspace for thirty days (up to 10 minutes a day). The control condition will be treatment as usual. At baseline and four weeks later, the following key outcome dimensions will be assessed: self-rated (Beck Depression Inventory) and experts’ rated symptoms of MDD (Hamilton Depression Rating Scale); secondary outcome variables will be blood pressure, heart rate, and respiratory rate and changes in tobacco and alcohol consumption and medication as a proxy of perceived stress.

**Results:**

This study was funded in February 2021 and approved by the institutional review board on April 15, 2021, and it started in May 2021. As of December 2021, we enrolled 30 participants. The findings are expected to be published in spring 2023.

**Conclusions:**

We hypothesize that compared to the control conditions, individuals with MDD of the mobile app-condition will have both lower self- and experts’ rated symptoms of MDD and more favorable stress-related levels. While the risk for medical events is low, the immediate benefit for participants could be a decrease in symptom severity and reduction of the stress level.

**Trial Registration:**

Clinical Trials.gov NCT05060393; https://clinicaltrials.gov/ct2/show/NCT05060393.

**International Registered Report Identifier (IRRID):**

DERR1-10.2196/33423

## Introduction

Major depression disorder (MDD) is a global, serious, and life-shortening disease, affecting about 300 million people worldwide and the main cause of disability-adjusted life years caused by mental diseases [1-3]. The limited capacities in ambulatory care and the increasing mental health needs request innovative, cost-effective interventions [4,5]. During the last decade, research has focused on cost-effective internet-based interventions as add-ons to existing services for individuals with MDD. Such interventions can potentially reduce the gap between the need and provision of psychiatric treatments, though being effective [6-8]. Recently, the need for remote psychological, psychiatric, and internet-based interventions in the mental health sector further increased during the worldwide COVID-19 pandemic and the health-related lockdown measures [9-11]. Accessible strategies can be an effective option to enhance mental health response capacity [12,13].

Mindfulness-based interventions (MBIs), such as mindfulness-based stress reduction or mindfulness-based cognitive therapy, have received considerable attention in modern psychotherapy because of emerging evidence regarding their efficacy in different clinical populations [14-19]. MBCT can both decrease depressive symptoms of a current depressive episode and avoid relapses in individuals with MDD [20-23]. The effects are comparable to those of other cognitive behavioral therapies [24-27] and showed favorable effects if used as an adjunct therapy to treatment as usual (TAU) [28-31]. There is growing evidence that web- and computer-based stress interventions can be effective in reducing stress, depressive, and anxiety symptoms [32-34].

In a study comparing internet-based psychological interventions in primary care, MBIs were effective in both decreasing depressive symptoms and increasing well-being compared to healthy lifestyle psychoeducational program of positive affect promotion [4] and compared to the waitlist control group [35].

As regards mobile applications, these may contribute to closing the treatment gap for depression by reaching large populations at relatively low costs [36]. There is evidence of positive effects of mindfulness-based mobile applications on well-being, stress level, affect, work engagement, irritability, mind wandering as well as sleep quality in different study populations [37-43]. Eight randomized controlled trials using a mindfulness-based app as an intervention group are reported in more detail in Table 1. The intervention period differed from 14 days up to eight weeks (median 28 days).

**Table 1:** Summary of randomized control trials using mindfulness-based mobile applications.

Author/year	Intervention (intervention group)	App	Control group	Study population	Outcome/psychometry	Results
O`Donnell [37], 2020	10 min a day over 30 days	Insight Timer	Wait-list control group	General adult population	Generalized Anxiety Disorder 7 (GAD-7); well being	Not available yet
Huberty [38], 2019	10 min a day over 56 days	Calm	Wait-list control group	College students	Stress, well-being, mindfulness, sleep, alcohol consumption, physical activity	Significant effects in all measured parameters in the intervention group
Möltner [39], 2017	Daily over 14 days	7Mind	Waitlist control group	Employees	Mindfulness, work engagement, job satisfaction, emotional exhaustion, emotional intelligence, innovation and creativity, and self-efficacy.	Significant effects in all measured parameter in the intervention group
Economides [40], 2018	10 min a day over 14 days	Headspace	Audiobook about mindfulness	General adult population with no history of psychiatric disorder	Stress-level, irritability, affection/Stress Overload Scale, Scale of Positive and Negative Experience (SPANE)	Significant reduction of stress level, irritability, significant changes in SPANE score in the intervention group
Howells [41] 2015	10 min a day over 14 days	Headspace	Catch Notes	Facebook and LinkedIn users	The Satisfaction with Life Scale, Flourishing scale, Positive and Negative Affect Scale, Center for Epidemiologic Studies Depression Scale (CESD)	Significant increase in positive effects in the intervention group
Bennike [42] 2020	10-20 a day over 28 days	Headspace	Cognitive training	General adult population with no history of psychiatric disorder	Mind wandering, Sustained Attention to Response Task (SART)	Sign. reduction of mind wandering in the intervention group
Lim [43] 2015	21 days	Headspace	Cognitive training	Students	Compassion	(not significant) increase of compassion in the intervention group
Ly [44] 2014	3-30 min a day over 56 days	App developed for the clinical trial	Behavioral activation via an app developed for the clinical trial	Patients with MDD (Major depression d isorder)	Symptom severity (Beck Depression Inventory II [BDI-II], Patient Health Questionnaire-9 [PHQ-9])	No significant differences between the intervention and the control group

In 4 of the 8 studies, the mindfulness-based intervention was delivered via the app Headspace, probably due to previous findings and a high score in the mobile application rating system [45], making this mobile app promising for further research. The application was brief and easy to use, free to download, and accessible via smartphones globally [41]. Users who sign up for the free trial have access to one of three guided foundation courses, titled “Basics.” There are 10 free sessions in each “Basics” course.

However, and surprisingly, from the reviewed studies, only one study targeted 40 individuals with MDD [44]; the results indicate comparable effects of a mindfulness-based app and behavioral activation via a major depression app.

Given this background and given the scarcity of app-based MBIs in individuals with MDD, the aim of this study is to examine the effects of a mindfulness-based mobile application (Headspace) on the symptom severity and physiological effects in participants with MDD in the real daily clinical practice as an adjunct therapy to the TAU.

The following hypotheses are formulated: first, following Howells et al [41], we assume that compared to a control condition, individuals in the intervention group will achieve a decrease in depressive symptoms. Second, following Huberty et al [38] and Economides [40], we expect a reduction of the stress level in the intervention group compared to the control group. For the following reasons, we hold that this study is of both practical and clinical importance. First, apps allow an individually tailored and self-paced intervention to improve symptoms of MDD and stress; second, such interventional add-ons improve individuals’ self-management and self-responsibility; third, interventional add-ons are easy to use, follow, complete, and monitor, allowing the user to get motivational-enhancing feedback; fourth, such apps provide easy-to-manage data to further improve the dialogue between the user and health care experts; fifth, once a person is accustomed to using the app, the odds are much higher than the person continues to use the app and the appropriate exercising long term.

## Methods

### Aims

The aim of this study is to assess the efficacy of a mindfulness-based mobile application (Headspace) on the symptom severity and the stress level measured by physiological parameters in patients with MDD when compared to a control group. We assume a decrease of the symptom severity, measured by the Beck Depression Inventory II (BDI-II) and Hamilton Depression Rating Scales (HDRS), in the intervention group compared to the waitlist control group. Furthermore, we hypothesize that the stress level assessed by heart and respiratory rate as well as the blood pressure will be lower in the intervention group compared to the control group.

### Study Design

This study will be a single-center, open-label and randomized wait-list-controlled trial, which will take place at the University Psychiatric Clinics (UPK) Basel (Switzerland).

**Figure 1:**
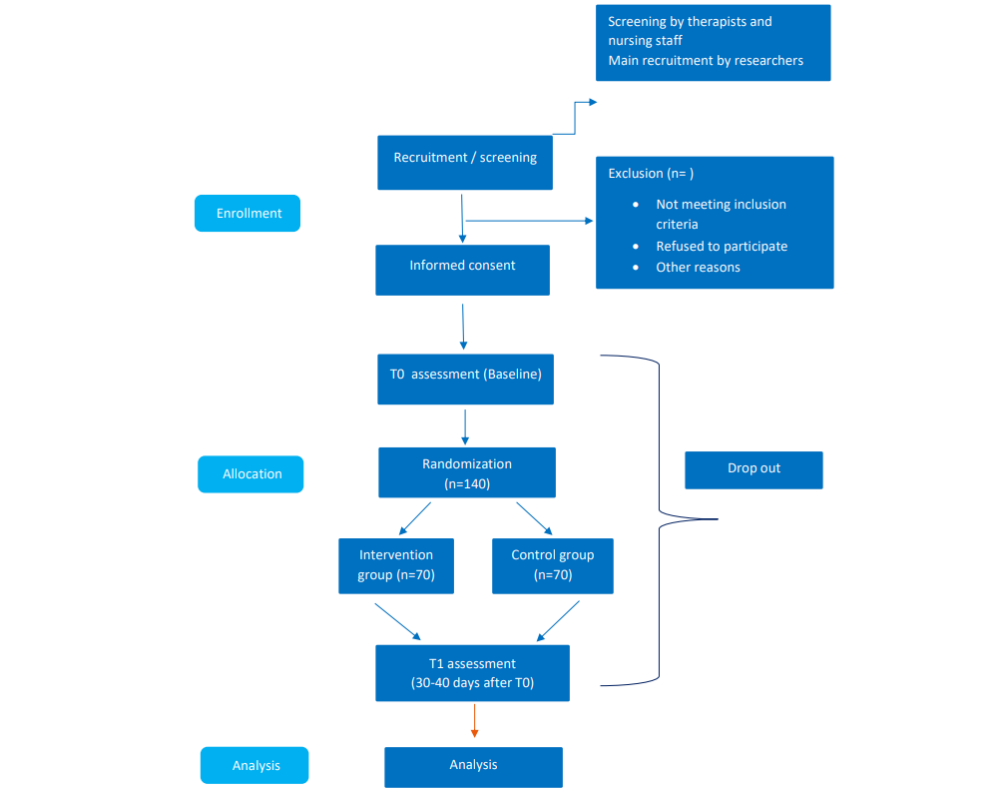
Flowchart study design.

Participants will be randomly allocated to two conditions: (1) MBI mobile application Headspace+ TAU (intervention group) and (2) waitlist + TAU (control group). They will be assessed at baseline and 30 days after baseline (study end). The study flowchart is presented in Figure 1 The study started in May 2021.

The study protocol will be introduced to all participants, the name of the mobile application is not to be communicated in the first step. Previous experience with mobile applications for health will be assessed in all subjects prior to randomization. Randomization is performed by the project leader by using a computer-based randomization algorithm, performing the randomization allocation (ratio 1:1). Each new participant of the study will receive a participant number that corresponds to the following treatment number.

At baseline, participants will complete a series of questionnaires covering sociodemographic and treatment-related information, along with a self-rating (ie, BDI-II) of current symptoms of MDD. An expert will perform a thorough clinical-psychiatric interview and rate participants’ symptoms of depression (ie, HDRS). To assess the current psychophysiological stress response at baseline conditions, a nurse will measure participants’ heart and respiratory rate and blood pressure.

Next, participants will be informed about the study condition assignment. Participants of the control condition will have no additional task to their TAU instruction. Participants of the intervention group will be introduced to the Headspace app and its handling. The necessary timeframe of the baseline assessment will be around 60 minutes.

The intervention group will be required to download the mobile application Headspace and start their daily usage in addition to their TAU (as explained in the “Intervention” section).

Regarding the control group, there will be no intervention planned. The control group will receive the same TAU as the intervention group during inpatient treatment. Participants of both groups will be supported in their outpatient treatment after discharge as usual. Compared to the treatment group, the control group will not be told about the application and its use but will be held on a waitlist. The control participants will be instructed to undergo their TAU (eg, private practice therapist), whereby they will be asked to come to the visit at measurement time T1 after 30 days (see the time points in Table 2).

At T1, both groups will undergo the same procedures as they did at baseline (maximum 60 min). Following the visit, the name of the mobile application will be communicated to the participants of the control group so they can use it after the end of the study as needed.

**Table 2:** Study timeline.

	Time points (days)		
		Baseline (T0)	T1
	–7 to 0	0	+30
Discharge (if inpatients)	√		
Inclusion/ exclusion criteria checked	√		
Signed written informed consent	√		
Medical history, medication	√		
Participant characteristics	√		
Randomization	√		
Visit T0 (baseline)		√	
Visit T1 (study end)			√

### Participants and Recruitment

In total, 140 patients should be recruited (70 for the intervention and control group each).

Inclusion criteria: inpatients or partial inpatients (day clinic, at least 18 years old, before discharge to ambulatory care as well as outpatients, all diagnosed with MDD according to the International Classification of Diseases. All participants must have a smartphone and be generally willing to download and use a mindfulness-based app for at least 30 days.

Exclusion criteria: Acute suicidality, dementia, acute substance dependency, psychotic, schizophrenic, or schizoaffective disorders, serious health conditions like unstable cardiovascular, heart, lung, endocrine or neurological disorders, and momentary use of Headspace.

The study will be brought to the attention of potentially eligible patients of the UPK through their health care providers at UPK and through the study flyer. If they show interest in participating, the patients will receive a document with all the required information regarding the study in order to give their informed consent. After the patients sign the form of consent, it will take between 1 to 7 days until they will get scheduled for their first appointment. This will give them an adequate amount of time to consider their participation carefully. Participants will be informed about the scope and procedure of the study. Their written informed consent will be obtained.

The participants can be withdrawn from the project for any of the following reasons:

1. At their own request (withdrawal of consent)
2. In case of nonadherence to the study protocol (if participants use the app on less than 25 days or if they use other courses beyond the basic course)
3. If one or more exclusion criteria appear within the time of observation

If a participant wants to discontinue participation, this will be accepted without the need for reasons. However, the participant will be asked for potential reasons for discontinuation to document it for further analysis. The date and, if available, the reason for the withdrawal will be documented, and the participant‘s data will be destroyed.

### Intervention

The intervention will consist of the download and the daily use of the mobile application Headspace in addition to the TAU. The participants will use the course basics of the application, which offers 10 guided meditation sessions. The duration of each session can be done in 3, 5, or 10 minutes. The study foresees that the course will be conducted in three series. The participants will make the first cycle of the course with 3 minutes per session, the second with 5 minutes, and the last one with 10 minutes per session. The participants will do one session a day, which adds up to 30 days of using the mobile application. The patients need no previous knowledge of mindfulness exercises or meditation. The course introduces and guides the patients in and through different meditation techniques. After 30 days of using the application, the patients will be examined at T1. In addition, at T1, all participants of the intervention group will be asked for short feedback about the intervention (“comment/feedback to the app use”).

To monitor the adherence to the protocol and keep participants engaged in using the app daily, participants of the intervention group will be contacted weekly via email or phone (as they prefer) and reminded of the daily use of the app.

### Primary Outcomes

The BDI-II and HDRS total scores, both continuous variables, will be used as the primary outcome measure of depression severity; BDI-II and HDRS are commonly used psychometric instruments with broad applicability in research and clinical practice [46,47].

The BDI-II is a 21-item self-reporting questionnaire. It evaluates the severity of depression in normal and psychiatric populations [32]. The BDI-II total score can be obtained by adding up the answers, from a minimum of 0 to a maximum of 63 points.

The HDRS is the most widely used rating scale for depression severity. However, the 21-item version is most commonly used for the reason that the last four items (diurnal variation, depersonalization/derealization, paranoid symptoms, and obsessional and compulsive symptoms) should not contribute to the total score. This study will be using the 17-item rating scale as well. The total score can be acquired by adding up the answers. It can range from a minimum of 0 to a maximum of 52 points.

The possibility of relapse or rehospitalization between the two assessments will be assessed at T1.

### Secondary Outcomes

Stress levels will be examined by autonomous nervous system parameters: resting heart rate, blood pressure as well as respiratory rate. The resting heart rate, as well as the blood pressure, will be measured by using a blood pressure monitor provided by the clinic. The respiratory rate will be assessed by counting the breaths taken per minute.

The same procedures will be used for all study participants to ensure that the data from all individuals will be comparable. All measurements will be taken in awake subjects in a sitting (slightly inclined) position and in the same room. All subjects will be asked to breathe normally and not move for five minutes to reduce the impact of pretest movements.

Furthermore, all current medications (inclusive anxiolytics and sedatives), general and momentary consumption of alcohol, as well as the consumption of nicotine, in particular pack-year and amount of daily smoked cigarettes, will be assessed.

### Data Analysis

Basic hypothesis:

- H1: Decrease (or lower increase) of the BDI-II and HDRS sum scores at T1 compared to baseline in the intervention group compared to the control group.
- H0: The changes in the BDI-II and HDRS sum scores between baseline and T1 do not differ between the intervention and the control group.

For the statistical analysis, we will use tools provided by the software packages IBM SPSS statistics and R (R Core Team). The changes in the symptom severity, as well as physiological parameters between baseline and T1 measurements, will be assessed. When comparing the change scores in the intervention group with those of the control group, typical hypothesis-testing, including mixed linear models, will be used, as provided by the software packages IBM SPSS statistics and R with a significance level of .05.

Sample size estimation: The estimation is based on an expected effect size of Cohen *d*=0.50, which is based on prior published studies of mindfulness-based apps. When setting the significance criterion α=.05 and β=.2 (statistical power 80%), the required sample size for a two-sample *t* test is 64 for each group. The dropout rate is expected not to exceed 10%, resulting in 140 patients overall.

### Ethics Considerations and Approval

This study will be conducted according to the international standards of the Declaration of Helsinki and subsequent amendments. This trial will be performed in compliance with the study protocol and with good clinical practice guidelines.

The study protocol has been approved by the Swiss Ethics Committee (North/Central Switzerland-EKZN; 2021-00452) and follows the guidelines of the Declaration of Helsinki and current Swiss legislation on privacy and data protection. The study participants will sign the written informed consent form before randomization. Participants will be provided with detailed information about the study and will be informed that they may leave the study at any time.

## Results

This study was funded in February 2021 and approved by the institutional review board on April 15, 2021, and it started in May 2021. As of December 2021, we have enrolled 30 participants. The findings are expected to be published in spring 2023.

## Discussion

This study aims to assess the efficacy of a mindfulness-based mobile application, Headspace, on depressive symptoms and physiological stress levels in outpatient MDD patients. We hypothesize fewer relapses of depressive symptoms and a decrease of symptom severity in the group of MDD patients using Headspace for 30 consecutive days, compared to the waitlist group. There is great potential in the use of application-based meditation since it is easily accessible, easy to use, cost-effective, and, most importantly, could help improve patients’ well-being. However, there are some practical issues to mention in this study. One issue regards the duration of the effect; although the intervention of 30 days is within the range of other studies (median 28 days), we will not be able to measure and report middle- and long-term effects (eg, 6 months postintervention). If our hypothesis is confirmed, further studies are needed to investigate middle- and long-term effects, and implementation of MBI via an app in evidence-based care is needed. Furthermore, this study will use one single application (Headspace) as the intervention method. Further studies will be needed to compare the effects of Headspace to other interventions. Another issue to consider is patient compliance. Due to the fact that our participants will be in an inpatient setting and the intervention consists of using the application by themselves, it will be difficult to monitor the actual use of the application and, therefore, patient compliance.

Fortunately, there are no risks of medical events as well as no known risks of the intervention planned. The only potential and very low risk could be due to unauthorized data access; however, this risk will be minimized due to the adherence to the study protocol. Further, electronic data will be saved on password-protected computers that are only accessible by authorized personnel. All paper data will be stored on-campus in locked cabinets. Only authorized personnel will have access to the study data.

The potential immediate benefit for the participants consists of a decrease in symptom severity and prevention of depressive relapse without side effects. The long-term benefits are that patient care, especially in the outpatient setting or after discharge from inpatient treatment, will be easily augmented and improved. Moreover, the patients will be supported by an easily accessible mindfulness-based intervention. Such benefits may accommodate the issue of limited capacities in ambulatory care. With the prevention of depressive relapse, people with MDD get the chance to not only improve in symptom severity but also to live in their homes, with their families, go to school, or to work, accompanied by a tool for stress reduction.

## Abbreviations

BDI-II: Beck depression inventory II
HDRS: Hamilton Depression Rating Scale
MBI: mindfulness-based intervention
MDD: major depression disorder
TAU: treatment as usual
UPK: University Psychiatric Clinics
